# Overcoming Resistance to Combination Radiation-Immunotherapy: A Focus on Contributing Pathways Within the Tumor Microenvironment

**DOI:** 10.3389/fimmu.2018.03154

**Published:** 2019-01-31

**Authors:** Laurel B. Darragh, Ayman J. Oweida, Sana D. Karam

**Affiliations:** Department of Radiation Oncology, School of Medicine, University of Colorado, Aurora, CO, United States

**Keywords:** immunotherapy, radiation therapy (RT), myeloid derived suppressor cell (MDSC), regulatory T (Treg) cell, tumor microenvironment (TME), immunotherapy resistance, cancer associated fibroblast (CAF)

## Abstract

Radiation therapy has been used for many years to treat tumors based on its DNA-damage-mediated ability to kill cells. More recently, RT has been shown to exert beneficial modulatory effects on immune responses, such as triggering immunogenic cell death, enhancing antigen presentation, and activating cytotoxic T cells. Consequently, combining radiation therapy with immunotherapy represents an important area of research. Thus far, immune-checkpoint inhibitors targeting programmed death-ligand 1 (PD-L1), programmed cell death protein 1 (PD-1), and cytotoxic T-lymphocyte-associated protein 4 (CTLA-4) have been the focus of many research studies and clinical trials. The available data suggest that such immunotherapies are enhanced when combined with radiation therapy. However, treatment resistance, intrinsic or acquired, is still prevalent. Various theories as to how to enhance these combination therapies to overcome treatment resistance have been proposed. In this review, we focus on the principles surrounding radiation therapy's positive and negative effects on the tumor microenvironment. We explore mechanisms underlying radiation therapy's synergistic and antagonistic effects on immune responses and provide a base of knowledge for radio-immunology combination therapies to overcome treatment resistance. We provide evidence for targeting regulatory T cells, tumor-associated macrophages, and cancer-associated fibroblasts in combination radio-immunotherapies to improve cancer treatment.

## Introduction

Radiation therapy (RT) represents standard-of-care treatment for more than half of all cancer patients (1). RT was originally used for its ability to induce double-stranded DNA damage resulting in cell death via apoptosis, necrosis, autophagy, mitotic catastrophe, or replicative senescence (2, 3). But RT can also modulate the immune system and the tumor microenvironment (TME) in a dose-dependent manner (4–6). Our increased knowledge of the positive immune-modulating effects of RT has led to the development of novel combination therapies. Several preclinical studies have shown that combining RT with immunotherapy (IT) can result in better local and systemic tumor control (5). Combining RT with anti-CTLA-4 therapy (7–10), anti-PD-1 (11–13), or anti-PD-L1 therapy (14–16), with RT doses ranging from 2 to 20 Gy in single and fractionated regimens, has resulted in prolonged survival and reduced tumor growth in preclinical tumor models (17). Emerging data from clinical trials combining RT and IT have also shown promise (18–21). Most recently, a Phase II clinical trial in which patients with locally advanced non-small cell lung cancer (NSCLC) or metastatic disease were treated with RT followed by pembrolizumab (anti-PD-1) found that this combination prolonged overall survival by 19.8 weeks (NCT02407171). Administration of nivolumab (anti-PD-1) before RT in another Phase II clinical trial looking at advanced NSCLC was shown to increase the 18 months survival of patients by 29% (22). Similarly, RT increased the effectiveness of PD-L1 inhibition in a retrospective study of recurrent/metastatic nasopharyngeal carcinoma (23). Although combining IT with RT has shown promising improvements in survival in these clinical trials, patients eventually relapse, and durable responses are rare (24). Several parameters can influence the response to IT and RT combinations, including RT dose, sequencing, and tumor oncogenic and immune composition. This variable success rate is thought to be caused by resistance—regrowth of the tumor—and is still common in most patients treated with radio-immunotherapy as some cancers like head and neck squamous cell carcinoma have a low response rate of 13% (25). By considering the cancer tumor microenvironment (TME) and its components, and how to specifically modulate them with RT and IT, we can potentially determine how to override resistance to radio-immunotherapy and improve outcomes.

**Figure 1:**
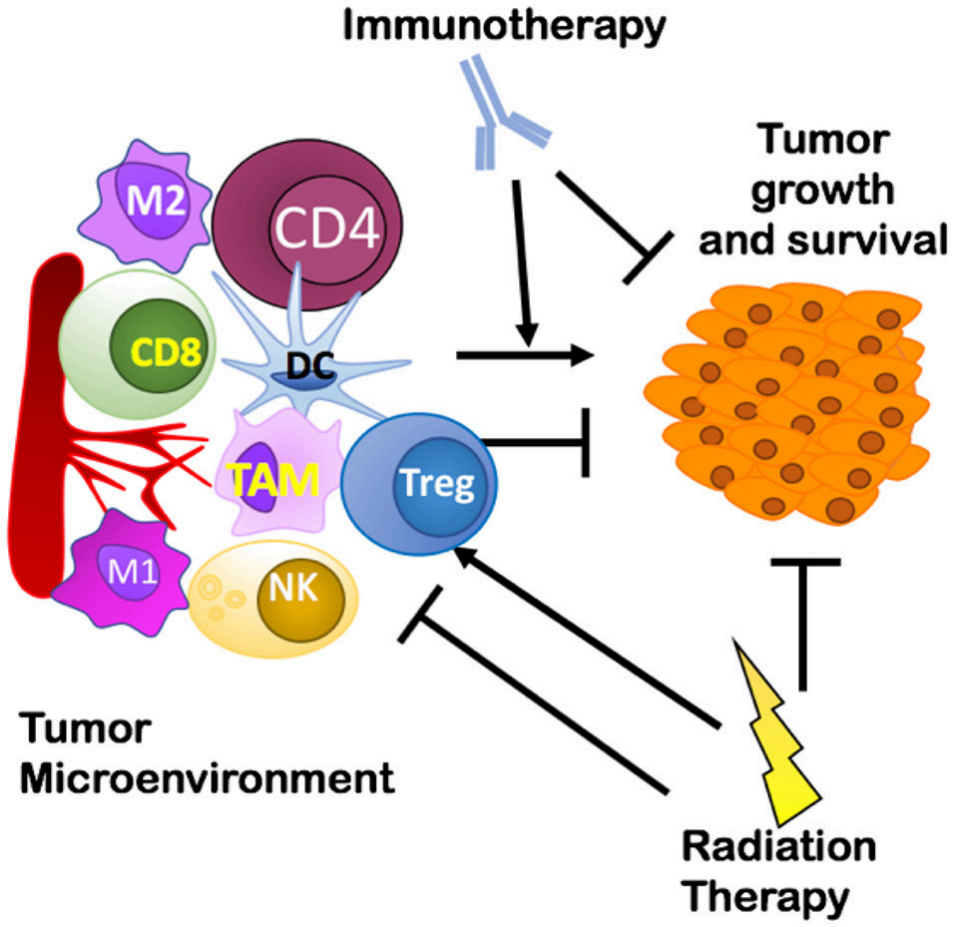
Overview of the interplay between a tumor and its microenvironment and potential targets of immunotherapy and radiation therapy covered in this review. Treg, Regulatory T cells; TAM, Tumor Associated Macrophages.

Various elements of the TME can prevent effective lymphocyte priming, reduce immune cell infiltration, and suppress effector cell function that can lead to a failure of the host to reject tumors (26). These elements identify several potential mechanisms that could affect the efficacy of radio-immunotherapy: suppressive immune cells including regulatory T cells (Tregs), macrophages, or myeloid derived suppressor cells (MDSC); lack of antigen stimulation/co-stimulation for dendritic cells (DCs) leading to inadequate T cell priming; physical barriers such as a thick extracellular matrix (ECM) produced by fibroblasts around tumor tissues preventing immune cell entry into tumors; and exhausted or short-lived activation of antigen-specific cytotoxic CD8^+^ T cells through activation of immune checkpoints like PD-1. Although tumor-intrinsic factors also play an important role in mediating growth and survival of the primary tumor (27), the focus of this review is on how elements of the TME can impact treatment outcomes, how RT modulates the immune TME, and potential immunotherapies that could improve RT's effects (as shown in Figure 1). This will provide a foundation for developing rational targeted ITs aimed at reducing the development of resistance when combined with RT. Further, it presents a rationale for shifting from broad targeting of immune checkpoint receptors to targeting of regulatory T cells, tumor-associated macrophages, and cancer-associated fibroblasts as specific targets for combination radio-immunotherapies. We conclude by suggesting that a thorough understanding of the biological pathways underlying known interactions between RT and various immune targets is and will continue to be invaluable for informing design of combination radio-immunotherapies to improve cancer treatment.

## Highlighting RT's Delicate Balance Between Promoting Immunosuppression and Tumor Cytotoxicity

To maximize the therapeutic ratio, it is important to establish a combination of ITs that activate pathways to promote anti-tumor immunity and effector T cell function while limiting pathways that mediate an immunosuppressive TME. Several mechanisms are involved in immune regulation and response to stress stimuli, including RT.

## RT Increases Type I IFN Secretion via STING Activation: A Dichotomy Between Dendritic Cell and MDSC Recruitment Determines the Therapeutic Response to Radiation

**Figure 2:**
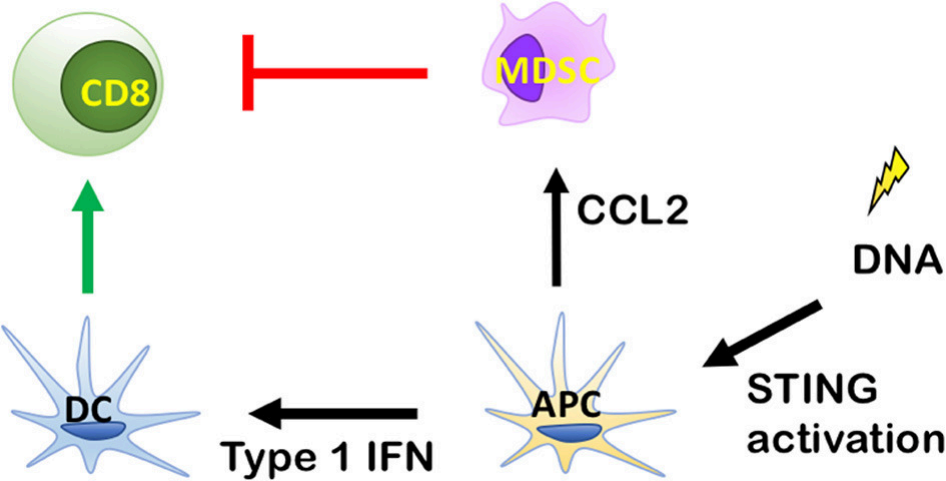
Role of the stimulator of interferon genes (STING) signaling pathway in antitumor immunity. By inducing Type I IFN release from antigen presenting cells (APCs), radiation therapy (RT) can enhance antigen uptake by specialized dendritic cells (DCs) known as Batf3 DCs. This stimulates maturation of DCs and the cross-presentation of tumor associated antigens (TAA) to CD8^+^ T cells, which exhibit antitumor immunity after proliferation and infiltration into tumor microenvironment. DNA from tumor cells is recognized by cytosolic DNA sensor cGAS to produce cGAMP for STING activation and cytokine production, which stimulate the maturation of DCs and stimulate the cross-presentation of TAA to CD8^+^ T cells, which exhibit antitumor immunity after proliferation and infiltration into the tumor microenvironment.

When RT induces tumor cell death, DNA from dying tumor cells is delivered to antigen presenting cells (APCs), most notably CD11c+ dendritic cells (DCs). In this process DCs are stimulated to present antigens, and express costimulatory molecules (28). A critical mediator of DC function is the stimulator of interferon genes (STING). STING pathway activation (Figure 2) occurs when DNA from tumor cells taken up by APCs is sensed by cyclic-GMP-AMP (cGAMP) synthase, which interacts directly with STING to induce a conformational change leading to translocation of STING from the endoplasmic reticulum to perinuclear vesicles (29). Inside the nucleus, STING recruits and phosphorylates TANK-binding kinase 1 (TBK1), which activates interferon regulatry factor 3 (IRF3). Finally, IRF3 induces expression of Type I IFNs (30). Type I IFN release from APCs facilitates the ability of Batf3 DCs to take up antigen (31). This stimulates maturation of DCs and cross-presentation of tumor associated antigens (TAA) to CD8^+^ T cells, which mediate antitumor immunity after proliferation and infiltration into the tumor microenvironment.

Type I IFNs induced by RT include IFN -α, -β and the less studied IFN-τ, -ε, -κ, and -ω. Expression of Type I IFN and Type I-inducible genes is associated with T cell-infiltrated tumors (32, 33). In addition, Type I IFN expression can be induced by RT. Burnette et al. showed increased Type I IFNs in a melanoma cancer model (B16-SYI) after 20 Gy of local RT (34). Knockdown of IFN-β receptor (IFN-α receptor 1) in B6/IFNAR1 KO mice abolished RT's ability to reduce tumor growth in this model. Lim et al. showed similar findings with a dose of 15 Gy (35). These data suggest that Type I IFNs, specifically IFN-β, may be key targets by which RT modulates the TME.

Deng et al showed that innate immune sensing following RT is predominantly mediated by a STING-dependent mechanism (31). The study demonstrated that cGAS- and STING-dependent cytosolic DNA sensing in DCs is required for type I IFN induction after RT and that adding the STING agonist cGAMP reduces radioresistance and enhances antitumor immune responses. However, the paradox of RT-mediated STING activation is that it can also recruit MDSCs (34, 36). While this could be an RT dose-dependent phenomenon, the recruitment of MDSCs by RT can inhibit CD8^+^ T cells and DC activity, thus negating any benefit from activation of the Type I IFN pathway. This has been demonstrated in MC38 colon tumors where irradiation was shown to primarily increase monocytic MDSCs (Ly6c^hi^ CD11b+ cells) (36). In support of this being mediated via the STING pathway, tumor irradiation in STING KO mice led to a significant decrease in MDSC recruitment (36). This evidence supports STING as an initiating factor in MDSC recruitment. It is possible that STING-mediated RT effects are tumor-specific. Tumors that are poorly MDSC infiltrated and/or do not induce MDSC chemoattractants in response to RT may benefit from a STING agonist in combination with RT. In contrast, tumors that are MDSC rich and/or activate MDSC recruitment in response to RT may require strategies for targeting MDSCs. Combining MDSC targeting therapies with RT may not only enhance STING activation, but also increase Type I IFN production and recruitment of CD8^+^ T cells (37, 38).

A potential target through which RT increases MDSC recruitment is the monocyte chemoattractant CCL2. In the MC38 colon tumor model above, genetic knockdown of CCL2 yielded complete tumor eradication in 60% of irradiated mice further supporting MDSCs as a major driver of immunosuppression (36). Similarly, monoclonal antibodies against CCL2 led to tumor rejection in 40% of mice, but only when combined with RT (36). Anti-CCL2 antibody therapy combined with RT also resulted in an increase of CD8^+^ T cell activity, measured by INF-γ by Elispot assay (36). Antitumor immune-mediated effects of CCL2 genetic knockdown or anti-CCL2 antibody treatment were abolished when both CD8^+^ and CD4^+^ T cells were depleted (36). This evidence indicates that MDSCs block RT-induced T-cell anti-tumor activity via CCL2 and suggests this is a therapeutic target that could be manipulated to tip the balance in favor of dendritic cell recruitment.

Combining MDSC targeting therapies with RT may not only enhance STING activation, but also increase Type I IFN production and recruitment of CD8^+^ T cells (37, 38). For tumors where MDSCs play a prominent role, using RT with STING immunotherapies may not be sufficient. MDSCs may be able to block the positive effects of these therapies by inhibiting CD8^+^ T cell activity. For these tumors, adding anti-CCL2 antibodies to the treatment may be prudent.

In addition to MDSCs, M2 macrophages play a similar role in mediating immune suppression and resistance to RT (39). The IL-6/JAK/STAT3 pathway has been shown to polarize macrophages toward the pro-tumoral M2 phenotype through activation of STAT3, and anti-IL-6 immunotherapy increased the number of M1 polarized macrophages in a hepatocellular carcinoma mouse model (40). A review focused on the IL-6/JAK/STAT3 pathway, its role in cancer, and possible inhibitors of the pathway was recently published by Johnson et al. (41). The effects of targeting IL-6 with RT in a murine model of prostate cancer resulted in attenuation of angiogenesis, MDSC recruitment and decreased tumor growth (39).

Collectively, these studies highlight that it may be prudent to combine RT with immunotherapies that target MDSC and/or M2 macrophage recruitment and polarization to enhance anti-tumor immune responses. Some initial successes in targeting macrophages have been achieved. Anti-CSF1 immunotherapy, when used in combination with RT, prolonged survival in a glioblastoma (GBM) mouse model and significantly reduced RT-mediated macrophage recruitment to the tumor (42). Chloroquine, a common drug used to treat malaria, has also been shown to have anti-tumor effects via its ability to convert M2s into an M1 phenotype. Alone, chloroquine was able to reduce tumor burden in a murine melanoma and a hepatocarcinoma model (43). Since chloroquine was shown to be dependent on T cells for its effects (43), it might induce an even larger reduction in tumor burden when combined with RT given RT's potent effect on increasing T cell infiltration. Finally, myeloid cells' activation status can be targeted for therapeutic development. One such example is CD40 a surface protein present on most APCs (44). When CD40 is activated on APCs by binding to CD40L, APCs are able to present antigens to T cells (45). Increasing antigen presentation with anti-CD40 therapy in combination with RT was shown to increase survival of a B-cell lymphoma mouse model to 100% when the study ended at 100 days post-tumor-inoculation (46).

## PD-L1-Dependent Resistance and PD-L1-Independent Resistance: How CD8^+^ T Cells Negatively Regulate Their Own Activation by IFN-γ and CCL22 Secretion

**Figure 3:**
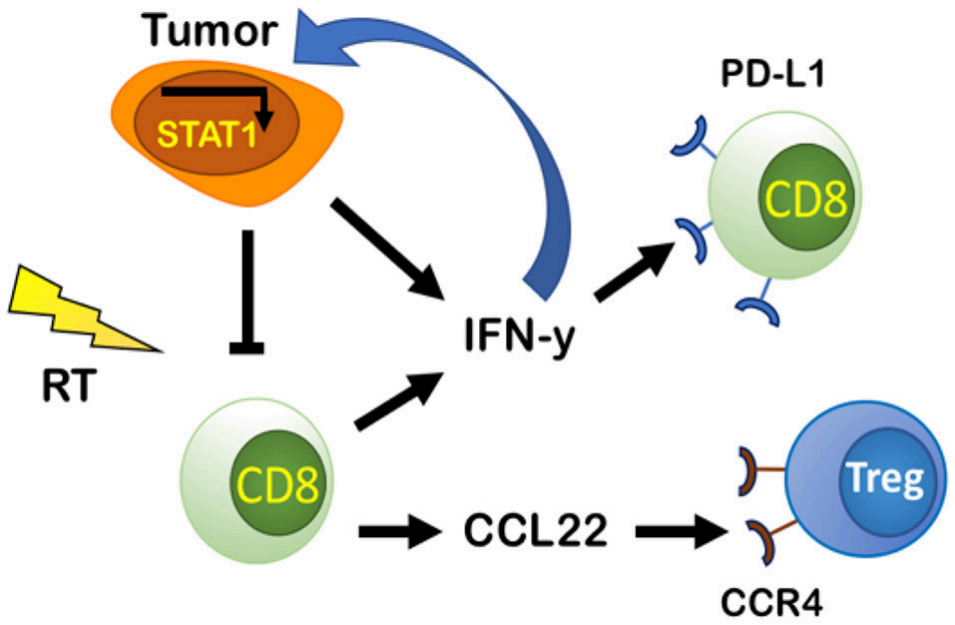
PD-L1-dependent and independent resistance by CD8 effector cells and tumor cells. Tumor cells secrete IFN-y and IFN-I that can bind to IFNGR and IFNAR on tumor cells and promote PD-L1-independent resistance through constitutive activation of STAT1. Tumor cells and CD8 effector cells produce and secrete IFN-y that increases PD-L1 in the TME and causes exhaustion of CD8 cells promoting PD-L1-dependent resistance. CD8 effector cells increase production of CCL22, a chemoattractant that binds to CCR4 on Tregs increasing their presence in the TME, thus decreasing CD8 effector cell activity.

RT's ability to recruit and activate CD8^+^ T cells by inducing secretion of chemoattractant molecules CXCL9, CXCL10, CXCL16, and CCL5 as a response to tissue damage is well-known (47–52). Despite this, resistance to RT still occurs. This is in part explained by CD8^+^ T cell exhaustion, which is characterized by increased expression of immune checkpoint receptors such as PD-1, resulting in PD-L1-dependent resistance (53). Tumor-intrinsic factors can determine the extent of PD-L1 expression in tumors treated with RT and chemotherapeutic agents (27), but it also increases in response to IFN-γ (53). Gajewski et al. found evidence that activated CD8^+^ T cells and their secretion of IFN-γ are responsible for promoting PD-L1 expression in the TME in a negative feedback loop *in vivo* (36). IFN-γ has been known for supporting an anti-tumor TME by promoting Th1 polarization, cytotoxic T cell activation, DC maturation (54), and increased CXCL9 secretion (55). But evidence now suggests that IFN-γ can also upregulate PD-L1 in the TME (53) (Figure 3).

IFN-γ's upregulation of PD-L1 has been shown in both murine and human tumor cell lines (56). The presence of both high CD8^+^ T cell infiltration and IFN-γ is required for PD-L1's increase in tumors. This has been demonstrated by comparing levels of PD-L1 and IFN-γ in WT mice and CD8 KO mice in multiple murine melanoma models (53). It has been postulated that IFN-γ upregulates PD-L1 expression through activation of IRF-1, an interferon regulatory factor with a binding site on the promotor of the gene coding for PD-L1 (57). IFN-γ's upregulation of PD-L1 supports the rationale for anti-PD-L1/PD-1 axis therapies in cancer therapy, but it also highlights why these therapies are only useful for a small portion of patients with high baseline levels of PD-L1 expression. Many tumors are devoid of T cells at baseline, and thus lack PD-L1 expression or effector T cells (Teff cells) that can be activated by anti-PD1/PD-L1 therapies (58). Combining such therapies with RT could be beneficial as RT increases PD-L1 expression and enhances infiltration of Teff cells (59).

Although combining RT and PD-L1 therapy has improved outcomes in more patients than anti-PD-L1 treatment alone, emerging data suggest that resistance still develops (24). In preclinical models, Benci et al. identified a novel role for INF-γ and Type I IFNs in PD-L1-independent resistance and showed that targeting IFN-γ/Type I IFNs resulted in decreasing T cell exhaustion (60). To determine if IFN-γ was responsible for resistance independent of PD-L1 expression, PD-L1 was deleted in tumor cells using CRISPR and PD-L1 was deleted in tumor associated macrophages (TAMs) or globally deleted with anti-PD-L1 therapy. The authors reported that IFN-γ expression was still able to induce resistance when PD-L1 was deleted, but when IFN-γ's receptor IFNGR and the receptor for Type I IFNs IFNAR were knocked out on tumor cells, exhausted T cells were significantly reduced and response to RT and anti-CTLA4 was enhanced (60). These data demonstrate that IFN-γ and Type I IFNs are responsible for promoting resistance to combined RT and anti-CTLA-4 treatment in a PD-L1-independent manner (60). Benci et al. further showed that this resistance is mediated by constitutive activation of STAT1 expression in tumor cells through genomic studies and effect studies involving STAT1 KOs combined with anti-PD-L1 treatment (60). Based on these results and the finding that IFN-stimulated genes are increased in patients who develop resistance to anti-PD-L1 therapy (60), screening patients for IFN-stimulated genes may determine if patients qualify for therapeutic combinations of RT, anti-PD-L1, or anti-IFN therapy.

CD8^+^ T cells can also regulate their own activity by recruiting Tregs through the CCL22-CCR4 axis (Figure 3). Gajewski et al. demonstrated that an increase in CCR4-expressing Tregs as a percentage of total immune cells was observed only when CD8^+^ T cells were present (53). In CD8 KO mice, Tregs represented a lower percentage of total immune cells (53). Through a series of experiments, they showed that secretion of CCL22 by CD8^+^ T cells recruits T cells and supports their proliferation without inducing T cell differentiation (53). Additionally, inhibition of CCR4 using the antagonist C021 prevented Treg accumulation in tumors (53). Targeting CCR4 could be a promising therapeutic target, especially in Treg enriched tumors. Such a therapy may have enhanced efficacy when combined with RT to induce Teff cell infiltration.

## RT-Induced Adenosine: Shifting the TME From Dendritic Cell Recruitment Toward Treg- and M2- Mediated Immune Suppression

**Figure 4:**
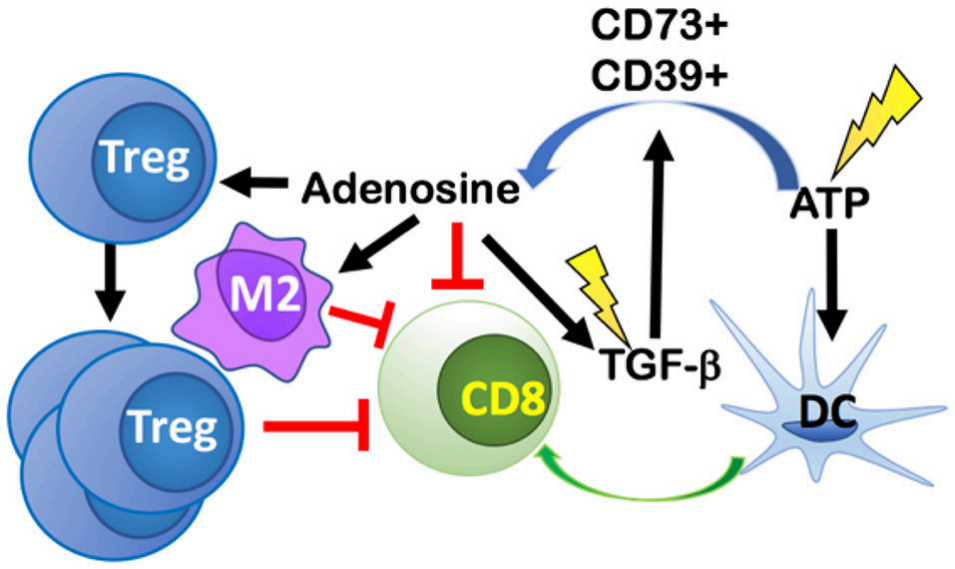
RT-induced cancer cell death leads to release of ATP that both recruits and activates dendritic cells (DCs) thus initiating antitumor immune responses. ATP is rapidly catabolized into adenosine in the TME by CD39 and CD73 expressed on tumor cells, stromal cells, and immune cells. Local accumulation of extracellular adenosine suppresses DCs and CD8 T cells, while promoting proliferation of Tregs, M2 polarization, and increasing the release of TGF-β into the TME. RT, can also directly activate TGF-β via activation of reactive oxygen species (ROS). The increase in TGF-β promotes more adenosine formation in a positive feedback loop.

Immunogenic cell death resulting from tumor irradiation alerts the immune system to a potential threat via upregulation or release of DAMPs, including adenosine triphosphate (ATP). The dose-dependent release of ATP as a result of RT-induced cancer cell death (61), can recruit and activate DCs to uptake tumor antigens and cross-present them to naïve T cells, thus initiating antitumor immune responses (62) (Figure 4). However, ATP is rapidly catabolized into adenosine in the TME by the ectoenzymes CD39 and CD73 expressed on tumor cells, stromal cells, and immune cells, primarily, Tregs and Th17 cells. CD39 hydrolyzes ATP to ADP, and ADP into AMP, and then CD73 converts AMP into adenosine (63). Local accumulation of extracellular adenosine suppresses DCs and Teff cells while promoting proliferation of Tregs, increases the expression of CTLA-4 and adenosine receptor 2A (A2AR) on Tregs, and enhanced the polarization of tumor-associated macrophages (TAMs) into an M2 suppressive phenotype (64, 65).

Conversion of ATP to adenosine can be induced directly by RT. One mechanism for this conversion is mediated via the induction of reactive oxygen species (ROS) by RT, which then converts pro-TGF-β into its activated form (66). TGF-β promotes TAM polarization into M2s and upon glucocorticoid induction, TGF-β modifies gene expression in M2 macrophages to express additional immune-suppressive genes like the one coding for IL-17 receptor (IL17RB) that promotes development of Th17 cells. TGF-β is also able to increase the expression of ectonucleotidases CD73 and CD39 on Th17 cells by downregulating zinc finger protein growth factor independent-1 (Gfi-1) and by inducing Stat3 expression, respectively (67). Taken together, TGF-β increases the number of Th17 cells and the expression of genes responsible for converting ATP into adenosine in Th17 cells.

Therapeutic targeting of A2AR, CD73, and TGF-β may shift the TME to a pro-ATP environment and reduce resistance to immunotherapy in the setting of RT. In preclinical animal models, targeting A2AR, the receptor for adenosine, with a pharmacological inhibitor SCH58261 led to a significant decrease in tumor growth and reduced Tregs while enhancing Teff cell activity in a spontaneous Cre/lox HNSCC model (68). Targeting A2AR alone with CPI-444 led to a significant reduction in tumor burden for mice implanted with MC38 tumors (69). Further tumor regression was achieved by the addition of anti-PD-L1 and anti-CTLA-4 treatment in both MC38 and CT26 tumors. The combination of CPI-444 and anti-PD-L1 in MC38 implanted mice led to a 50% eradication of the tumors (5/10) (69). Another way to reduce the effects of adenosine is to limit its production in the first place by targeting CD73. Targeting CD73 with an anti-CD73 monoclonal antibody (mAb), anti-CD73 decreased the tumor burden and increased the survival of mice with MC38-OVA tumor cells (70). This effect was even greater when combined with anti-PD-L1 and anti-CTLA-4 (70). Another group found that CD73 knockout mice had greater homing of Teff cells and that this effect was primarily driven by CD73 expression on Tregs (71). Although blocking production and direct action of adenosine has been shown to be effective, therapeutic strategies aimed at targeting TGF-β can be of more significant benefit in combination with RT. TGF-β increases the expression of both CD73 and CD39 and is responsible for promoting a variety of pro-tumor effects. There has been some hesitation in targeting TGF-β in the past because of the potential for cardiac toxicities, but new-generation small-molecule inhibitors have been shown to have limited side effects in clinical trials (72–74). The newly developed bifunctional fusion protein, M7824, TGF-β Trap (75) is another potential therapeutic target to combine with RT, as it simultaneously blocks the PD-L1 and TGF-β pathways and might yield increased response compared to monotherapy alone.

## Classic RT Anti-Tumor Effects Mediated Through ROS Have a Dark Side: Increasing Adenosine Through Treg Apoptosis and Creating a Hypoxic Immunosuppressive TME

**Figure 5:**
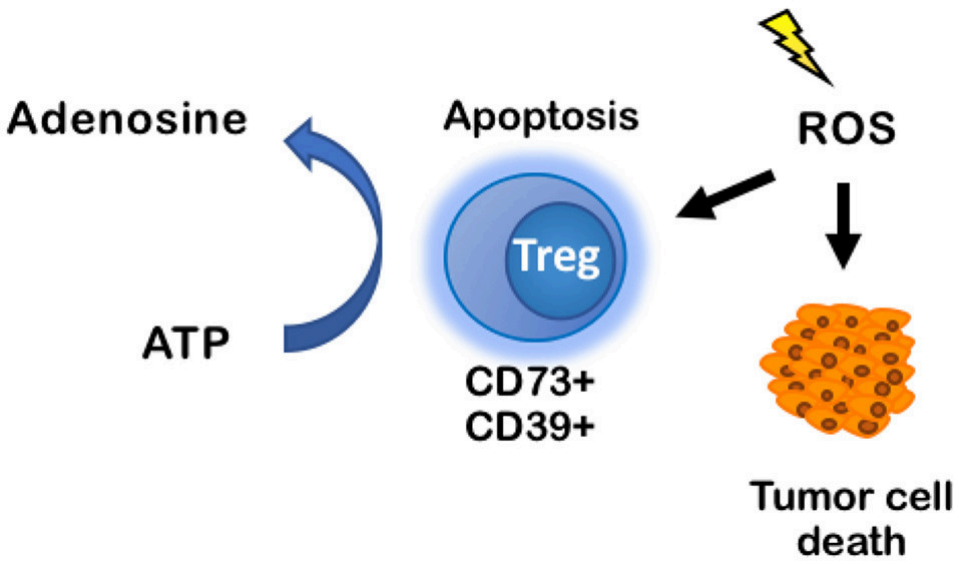
ROS induces apoptosis of CD73+CD39+ Tregs, increasing production of adenosine in the TME. ROS classically induces tumor cell death through DNA-mediated cell damage, but it also induces apoptosis in immune cells. When CD73+CD39+ Tregs undergo apoptosis, they produce high levels of ATP that are rapidly converted into adenosine by CD73 and CD39 on the Tregs cell membrane. Accumulation of adenosine promotes an immuno suppressive environment. ROS, reactive oxygen species.

RT is classically known to act on cancer cells by inducing apoptosis, senescence, and mitotic catastrophe through the production of reactive oxygen species (ROS) that, at high enough concentrations, can damage cells and cause double-stranded DNA damage (2, 3). It was thought that these effects would primarily affect tumor cells, causing tumor cell death, and—based on current understanding—increase tumor associated antigens for immune cell recognition. Recently, Zou et al. showed that within the immune TME, ROS resulting from RT induced apoptosis of Tregs driving increased immunosuppression. Their data support a hypothesis that apoptotic, but not proliferating, Tregs release high levels of ATP and subsequently metabolize ATP into adenosine because CD73 and CD39 are still metabolically active (76) (Figure 5). This fundamentally changes the current dogma of targeting all Tregs with immunotherapies. If Treg apoptosis is driving immunosuppression, an ideal immunotherapy would decrease Treg activity and proliferation, without inducing their apoptosis.

A more hypoxic environment will be less sensitive to the effects of RT (77), and many solid tumors are known to be more radioresistant in hypoxic regions. Although there is intrinsic hypoxia due to the nature of solid tumors, RT can worsen hypoxic conditions by increasing hypoxia-inducible factor-1α (HIF-1α). HIF-1α has been shown to cause radioresistance of endothelial cells (78), angiogenesis through expression of vascular endothelial growth factor A (VEGF-A) (79), malignant progression (79), and poor survival outcomes after RT treatment (80, 81). Upregulation of HIF-1α by RT can be a direct result of stabilizing HIF-1α in cancer cells (78, 79), or it can occur indirectly as RT increases TAMs, which also stabilize HIF-1α (82).

**Figure 6:**
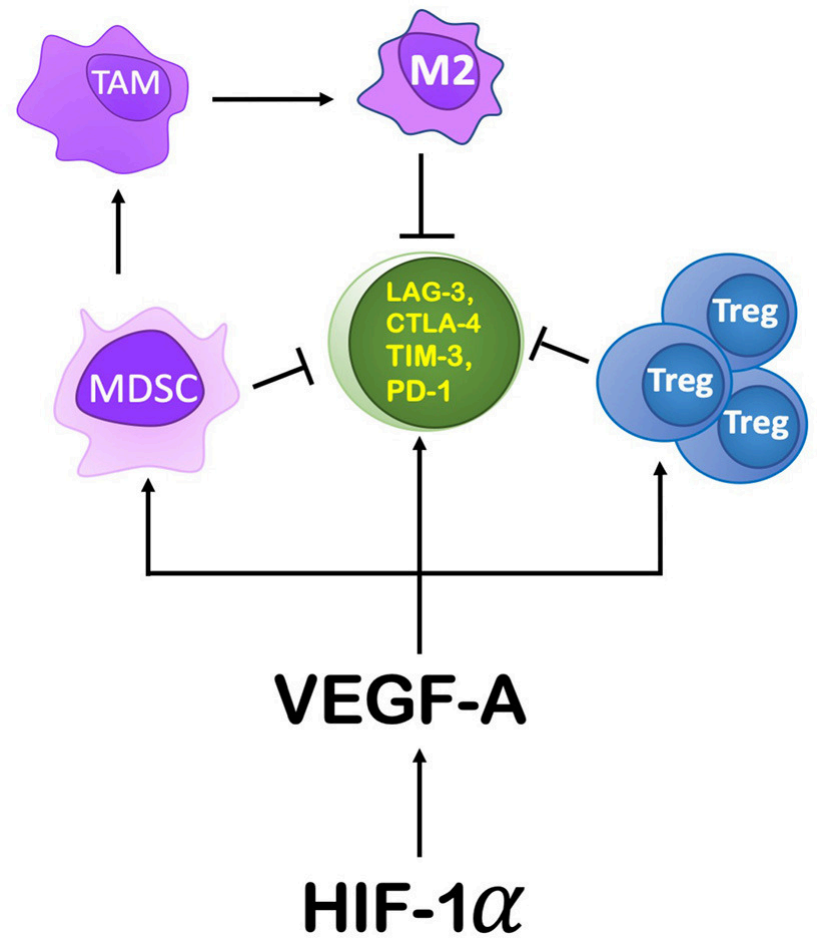
The role HIF-1α and its downstream components play in producing an immunosuppressive environment. HIF-1α's action on the TME is primarily through its induction of VEGF-A. VEGF-A drives immunosuppression by recruiting MDSCs, promoting proliferation of Tregs, and by increasing the expression of immune checkpoint inhibitor genes on CD8^+^ T cells. Increasing MDSCs in the TME leads to their conversion to TAMs, specifically an M2 polarization.

Within the TME, HIF-1α mediates immunosuppression by modulating specific immune cell functions (Figure 6). It modulates gene expression and cytokine production in MDSCs, thereby increasing their role in T cell suppression. HIF-1α inhibits myeloid cell differentiation through a VEGF-A mediated mechanism leading to accumulation of MDSCs (83, 84). Induced by RT, VEGF-A can also increase inhibitory receptors on CD8^+^ T cells (e.g., Tim-3, CTLA-4, PD-1, Lag-3) (85) as well as PDL-1 expression on tumor cells and MDSCs (86), thereby promoting T cell exhaustion and inactivity (85). Another mechanism by which RT-induced VEGF-A secretion can enhance a pro-tumor environment is through its influence on endothelial cells by inducing expression of CD95L (or FasL), the ligand for FAS (87, 88). In response to RT, expression of Fas can be induced by tumor cells secreting IL-10, prostaglandin E2, and VEGF-A (89). Fas can induce apoptosis of Teff cells, while sparing Tregs to support an anti-tumor environment (90).

HIF-1α represents an ideal target for reducing the immunosuppression driven by a hypoxic environment, but currently no drugs are approved for clinical trials in humans. Drugs designed in an attempt to target HIF-1α have had many off-target effects, including but not limited to inhibiting mRNA expression, protein synthesis, protein degradation, and DNA binding (91). In the future, more effective and specific inhibitors of HIF-1α will be developed. In the meantime, targeting VEGF-A may have some potential. There are several FDA-approved drugs that target VEGF-A, including the monoclonal antibody bevacizumab (66). Pre-clinical and clinical applications of these drugs have been well-described by others recently (92–94). Briefly, inhibiting VEGF-A appears to produce only a modest increase in survival for patients with a wide range of tumor types (95–99). These modest effects could be the result of indiscriminate administration of the drugs and/or parallel pathways of resistance. Combination approaches targeting both VEGF-A and HIF-1α axes or with cox-1 inhibitors as described in the next section could prove to be more beneficial than any single approach.

## RT's Remodeling of the Extracellular Matrix and Endothelial Cells: Promoting Fibrosis, MMP Activity, and FasL Expression

**Figure 7:**
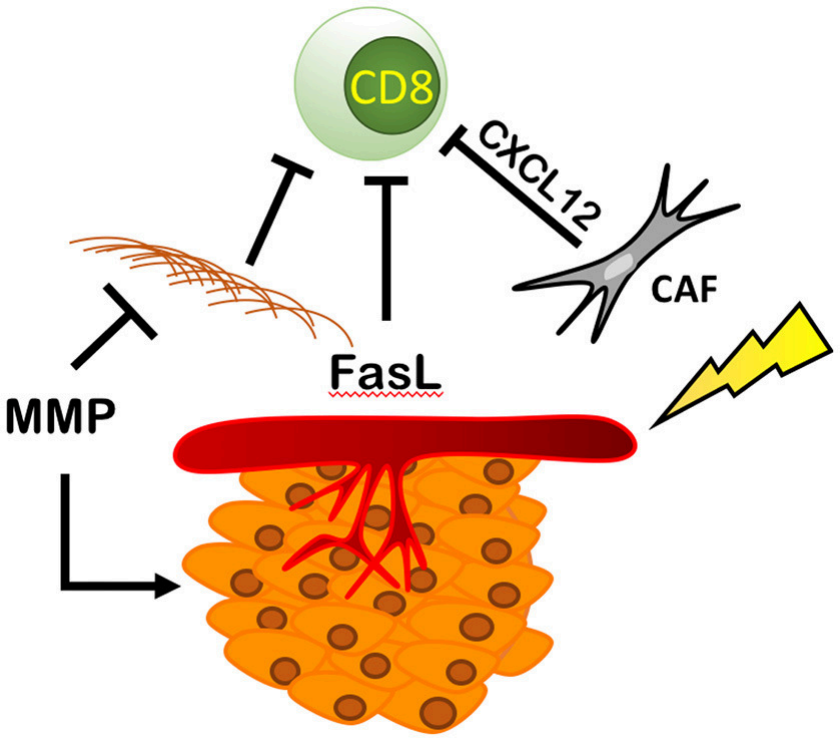
RT modulates fibroblasts, ECM, and endothelial cells resulting in an immunosuppressive environment. RT increases the number and activity of cancer associated fibroblasts (CAFs) increasing the production of CXCL12, blocking CD8^+^ T cell recruitment and increasing the amount of ECM proteins produced by fibroblasts physically blocking immune cells from entering the TME. This is countered by RT's ability to increase the expression of MMPs that break down the ECM, increasing cancer spread and metastasis. Finally, RT is able to increase the expression of Delta, Jagged, Notch, and FasL, thus reducing CD8^+^ T cell recruitment and promoting tumor growth and survival.

By increasing the number and activity of fibroblasts and MMPs, and increasing pro-tumoral endothelial cell function, RT can directly modulate the extra-cellular matrix (ECM) component of the TME (Figure 7). RT-mediated TGF-β signaling increases the number of cancer-associated fibroblasts (CAFs) or myofibroblasts in the ECM. These cells deposit type I, type II, type III, and type V collagen, fibronectin, and matrix metalloproteinases (MMPs) that regulate ECM homeostasis (100–102). CAFs also express fibroblast activation protein (FAP) (103) enhancing immunosuppression within the TME via CXCL12 (104), a chemokine that reportedly coats tumor cells and inhibits recruitment of T cells in the area (104) and reduces ECM-associated fibrosis (105).

RT can directly modulate endothelial cell function to inhibit Teff cell immune function and create a pro-tumoral TME. Upregulation of FasL, on endothelial cells has been shown to be a critical mediator of Teff cell inhibition in a variety of cancers (87, 88, 90). Fas can induce preferential apoptosis in Teff, while sparing CD25-expressing Tregs, favoring an immunosuppressive TME (90). Tumor-derived IL-10 and prostaglandin E2 can independently increase endothelial cell expression of Fas, and tumor derived VEGF-A is dependent on the presence of IL10 or prostaglandin E2 to further increase Fas expression (90). This explains why a blockade of FasL expression in different ovarian cancer cell lines by targeting VEGF-A was shown to be drastically enhanced when combined with COX1 inhibitors (90). VEGF-A's effects were dependent on the amount of COX 1 expression, implying that VEGF-A is necessary but not always sufficient to produce FasL (90). These results have been corroborated by findings in four distinct murine cancer models: ovarian, skin, colon, and renal cancer (90). Treatment of these tumors with anti-VEGF-A combined with a COX 1 inhibitor, salicylic acid, resulted in depletion of FasL expression on tumor endothelial cells, an increase in CD8^+^ T cells infiltrating the TME, and a reduction in tumor growth (90). Targeting VEGF-A alone has had modest effects on overall survival in clinical trials (92–94). Of note, aspirin has also been associated with prevention of colorectal cancer and a reduction in colorectal cancer mortality (106). Combining VEGF-A inhibitors with daily aspirin use may present a potential therapeutic combination to improve upon these modest anti-VEGF-A effects.

RT can also modulate the vascular TME to enhance tumor metastasis, which is in part mediated by upregulation of various genes involved in migration/metastasis. Tumor cell dissemination via blood vessels requires tumor cells to undergo transendothelial migration. This occurs at sites where leukocytes and macrophages are in direct contact with tumor cells and endothelial cells. The best studied proteins at these sites are Jagged, Delta, and Notch (107). Activated Notch1 has been shown to inhibit apoptosis and enhance radioresistance (108), while downregulation of Notch1 expression can induce radiosensitization and alleviate radiation-induced epithelial-mesenchymal transition (EMT) (109–111). Activation of the Notch signaling pathway upregulates E-selectin expression on endothelial cells that shields tumor cells in the blood stream from anoikis (112). In breast cancer models, inhibition of the Notch signaling pathway blocked macrophage-induced intravasation *in vitro* and the dissemination of tumor cells from the primary tumor *in vivo* (113). The association between EMT and radioresistance and the prominent role of Notch signaling as a driving force in the EMT process, suggest that Notch inhibition will result in radiosensitization of tumors that underwent EMT.

Targeting the fibrotic TME can be challenging. Kalluri et al. found that when myofibroblasts were eliminated from the TME using transgenic mice, cancer progression and outcome were worse (113). Deletion of myofibroblasts was also associated with a reduced Teff/Treg ratio and elevated CTLA-4 expression (113). This could be because certain structure is needed to allow for normal functioning of immune cells and to keep the tumor in place. However, some tumor types are known to be highly fibrotic, hence reducing, but not eliminating, fibrosis may be important for enhancing anti-tumor immunity. As treatment with RT can result in a significant increase in fibrosis in these tumors (114–118), it may also be important to use anti-fibrotic agents to reduce fibrosis to pre-RT levels. Some available drugs are being tried pre-clinically to reduce fibrosis. For example, Pirfenidone, which inhibits TGF-induced fibrosis by targeting the TGF-β1/Smad/CTGF pathway (119) has been shown to reduce RT-mediated fibrosis in a murine lung carcinoma model (120) and increase survival (121). Another avenue to increase T cell infiltration is targeting CXCL12 or its receptor, CXCR4. Targeting both has been shown to reduce RT-associated lung fibrosis (122), and anti-CXCL12 therapy alone increases T cell infiltration into tumors (104). Although directly targeting FAP+ cells represents an attractive therapeutic strategy, thus far targeting FAP alone has shown no benefit in clinical trials (115).

## Conclusion

There is now considerable evidence that single-agent immune therapies have limited response in various cancer sites (123–126). Radiation therapy has been shown to synergize with immune modulating therapy through several mechanisms including exposure of neo-antigens, STING activation and PD-L1 upregulation (31, 59, 127). In addition to synergy where each component contributes to tumor response, radiation therapy can transform tumors and sensitize them to immune therapies (7, 9, 12, 17). However, in both cases the response to combination RT and immune therapy can be transient. The challenge ahead is to determine why the combination of RT and immune therapy provides a durable response in some patients and a limited response in others. Specifically, future studies should focus on identification of how RT's paradoxical effects manifest in responders and non-responders. The response to RT and immune modulating therapy can be suppressed through additional mechanisms of immune-evasion and immune-suppression including chronic IFN-γ activation, conversion of ATP to adenosine, ECM remodeling and secretion of immunosuppressive factors that promote infiltration of Tregs, MDSCs and macrophages. These mechanisms are likely activated by tumor-intrinsic factors that should be identified and targeted to develop effective therapies. It is conceivable that such factors will affect RT response differently between patients with the same cancer type and across different cancer types. Therefore, identifying diagnostic biomarkers for these factors is an important next step. Tumor staining for PD-L1 expression has been successfully implemented in NSCLC and melanoma patients to identify candidates who will benefit from anti-PD-1/anti-PD-L1 therapy. Additional markers are warranted to identify candidates such as immune checkpoint receptors such as TIM-3, LAG-3, CTLA-4, as well as assessment of intratumoral Tregs, MSDCs and macrophages are warranted. Furthermore, assessment of secreted factors will be important for identifying patients who can benefit from therapies that target recruitment and homing of immune suppressive cell populations. Such factors include TGF-β, ATP, CCL2, CCL20, and CCL22. Another challenge of integrating RT with immunotherapy is identifying the RT dose and fractionation resulting in optimal synergy. Most evidence suggests that hypofractionated RT is better suited for integration with immunotherapy, but there is also evidence that conventional fractionation can achieve similar results. Consideration for when certain immune cell populations are more abundant may be beneficial in determining an optimal dosing schedule (128, 129). It is important to design clinical trials that address RT's effects on the TME, as well as dosing and fractionation when combined with immunotherapy. Selecting rational combinations of therapies based on both forward and reverse translation, rigorous preclinical studies, and careful analysis of trial specimens is needed to generate a mechanistic understanding of the effects of treatment on the tumor and the associated microenvironment.

## Author Contributions

LD, AO, and SDK contributed to the writing and editing of the manuscript. SDK is responsible for supervision of this work.

### Conflict of Interest Statement

The authors declare that the research was conducted in the absence of any commercial or financial relationships that could be construed as a potential conflict of interest.
